# Salicylates in Pleurisy

**Published:** 1890-05-03

**Authors:** 


### SALICYLATES IN PLEURISY.

Dr. Stiller recommends salicylate of soda in serous pleurisy, finding it useful not only in reducing the fever, but in aiding the absorption of the fluid, its diuretic action soon becoming evident when 3 to 4 grams (45 gr.-ʒi.) are taken daily. In empyema it is useless, so that Dr. Stiller believes it might be employed as a means of differential diagnosis between serous and purulent exudation, in conjunction, we would add, with Bacelli's test, or the transmission through pus, as approaching a solid medium in its density, of the whispered voice fremitus inaudible through a clearer fluid: a test which fails only in being occasionally simulated when thick fibrinous deposits are present.

